# Supplementary material for: P2 × 7 Receptor Inhibits Astroglial Autophagy via Regulating FAK- and PHLPP1/2-Mediated AKT-S473 Phosphorylation Following Kainic Acid-Induced Seizures
Source: Int J Mol Sci. 2020 Sep 4;21(18):6476. doi: 10.3390/ijms21186476 (PMC7555659; doi:10.3390/ijms21186476)
Supplement: Supplementary file 1 [file ijms-21-06476-s001.pdf]

Supplementary information

P2X7 receptor inhibits astroglial autophagy via regulating FAK- and PHLPP1/2-mediated AKT-S473 phosphorylation following kainic acid-induced seizures

Duk-Shin Lee[1] and Ji-Eun Kim[1,*]

[1]*Department of Anatomy and Neurobiology, Institute of Epilepsy Research, College of Medicine, Hallym University, Chuncheon 200-702, South Korea*

* Correspondence to: J.-E Kim, Department of Anatomy and Neurobiology, College of Medicine, Hallym University, Chunchon, Kangwon-Do 24252, South Korea; Tel: +82-33-248-2522; Fax: +82-33-248-2525; E-mail: jieunkim@hallym.ac.kr

Supplementary Table 1. Primary antibodies used in the present study

Antibody	Host	Manufacturer (catalog number)	Dilution used
AKT	Rabbit	Cell signaling (#9272)	1:1,000 (WB)<br>1:50 (IP)
AMPK	Rabbit	Abcam (ab3759)	1:1,000 (WB)
Bif-1	Rabbit	Cell signalling (#4427)	1:1,000 (WB)
ERK1/2	Rabbit	Biorbyt (orb160960)	1:1,000 (WB)
FAK	Rabbit	Proteintech (12636-1-AP)	1:1,000(WB)
GFAP	Mouse	Millipore (mab3402)	1:4,000 (IF)
GSK3β	Rabbit	Elapscience (ENT2082)	1:1,000 (WB)
HSP25	Rabbit	Enzo (ADI-SPA-801)	1:1,000 (WB)<br>1:500 (IF)<br>1:50 (IP)
Iba-1	Rabbit	Biocare Medical (CP 290)	1:500 (IH)
LAMP1	Rabbit	Lifespan (LS-B580)	1:1,000 (WB)<br>1:200 (IF)
mTOR	Rabbit	Cell signalling (2972S)	1:1,000 (WB)
NeuN	Guinea pig	Millipore (#ABN90P)	1:1000 (IH)
p-GSK3β-S9	Rabbit	Biorbyt (orb14745)	1:1,000 (WB)
p-AKT-S473	Rabbit	Cell signalling (#4060)	1:1,000 (WB)
p-AKT-T450	Rabbit	Cell signalling (9267S)	1:1,000 (WB)
p-AMPK-T172	Rabbit	Abcam (ab195946)	1:1,000 (WB)
p-ERK1/2	Rabbit	Bioss (bs-3330R)	1:1,000 (WB)
p-FAK-Y397	Rabbit	Cell signaling (#8556)	1:1,000(WB)<br>1:50 (IF)
p-FAK-Y576	Rabbit	Novusbio (NBP1-72238)	1:1,000(WB)
p-mTOR-S2448	Rabbit	Cell signalling (2971S)	1:1,000 (WB)
p-mTOR-S2481	Rabbit	Cell signalling (2974S)	1:1,000 (WB)
p-p70S6K-T389	Rabbit	Cell signalling (#9205)	1:1,000 (WB)
p-PDK1-S241	Rabbit	Cell signaling (#3061)	1:1,000(WB)

p-PI3K-Y458	Rabbit	Cell signalling (4228S)	1:1,000 (WB)
p-PRAS40-T246	Rabbit	Abcam (ab134084)	1:1,000 (WB)
p-PTEN	Rabbit	Cell signalling (#9549)	1:1,000 (WB)
p-Raptor-S792	Rabbit	Cell signaling (#2083)	1:1,000(WB)
p-Rictor-T1135	Rabbit	Cell signaling (#3806)	1:1,000(WB)
p-SP1-T739	Rabbit	Abcam (ab195733)	1:1,000 (WB)
p-ULK1-S555	Rabbit	Cell signalling (#5869)	1:1,000 (WB)<br>1:50 (IF)
p-ULK1-S757	Rabbit	Cell signalling (#14202)	1:1,000 (WB)
p70S6K	Rabbit	Proteintech (14485-1-AP)	1:1,000 (WB)
PDK1	Rabbit	Cell signaling (#3062)	1:1,000 (WB)
PHLPP1	Rabbit	Millipore (#07-1341)	1:1,000 (WB)
PHLPP2	Rabbit	Abcam (ab71973)	1:2,000 (WB)
PI3K	Rabbit	Cell signalling (4292S)	1:1,000 (WB)
PRAS40	Rabbit	Abcam (ab181408)	1:1,000 (WB)
PTEN	Rabbit	Abcam (ab32199)	1:10,000 (WB)
Raptor	Rabbit	Cell signaling (#2280)	1:1,000 (WB)
Rictor	Rabbit	Cell signaling (#2114)	1:1,000 (WB)
RIP	Mouse	Millipore (MAB1580)	1:5,000 (IF)
SP1	Rabbit	Elapscience (ENT3472)	1:1,000 (WB)
ULK1	Rabbit	Cell signalling (#8054)	1:1,000 (WB)
β-actin	Mouse	Sigma (A5316)	1:5,000 (WB)

Co-IP, Co-immunoprecipitation; IF, Immunofluorescence; WB, Western blot.

Fig. 1

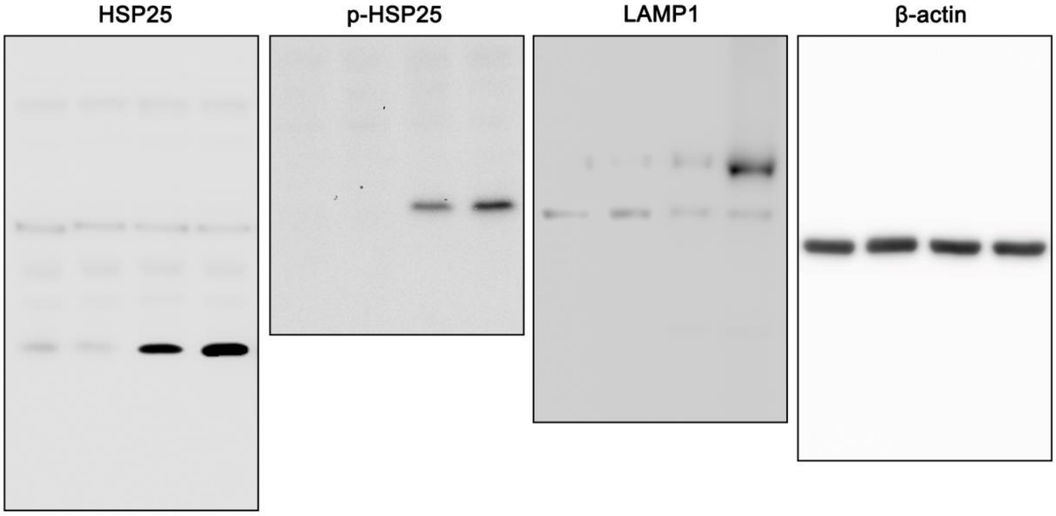

Supplementary Figure 1. Full-length gel images of Western blot data in Fig. 1.

Fig. 2

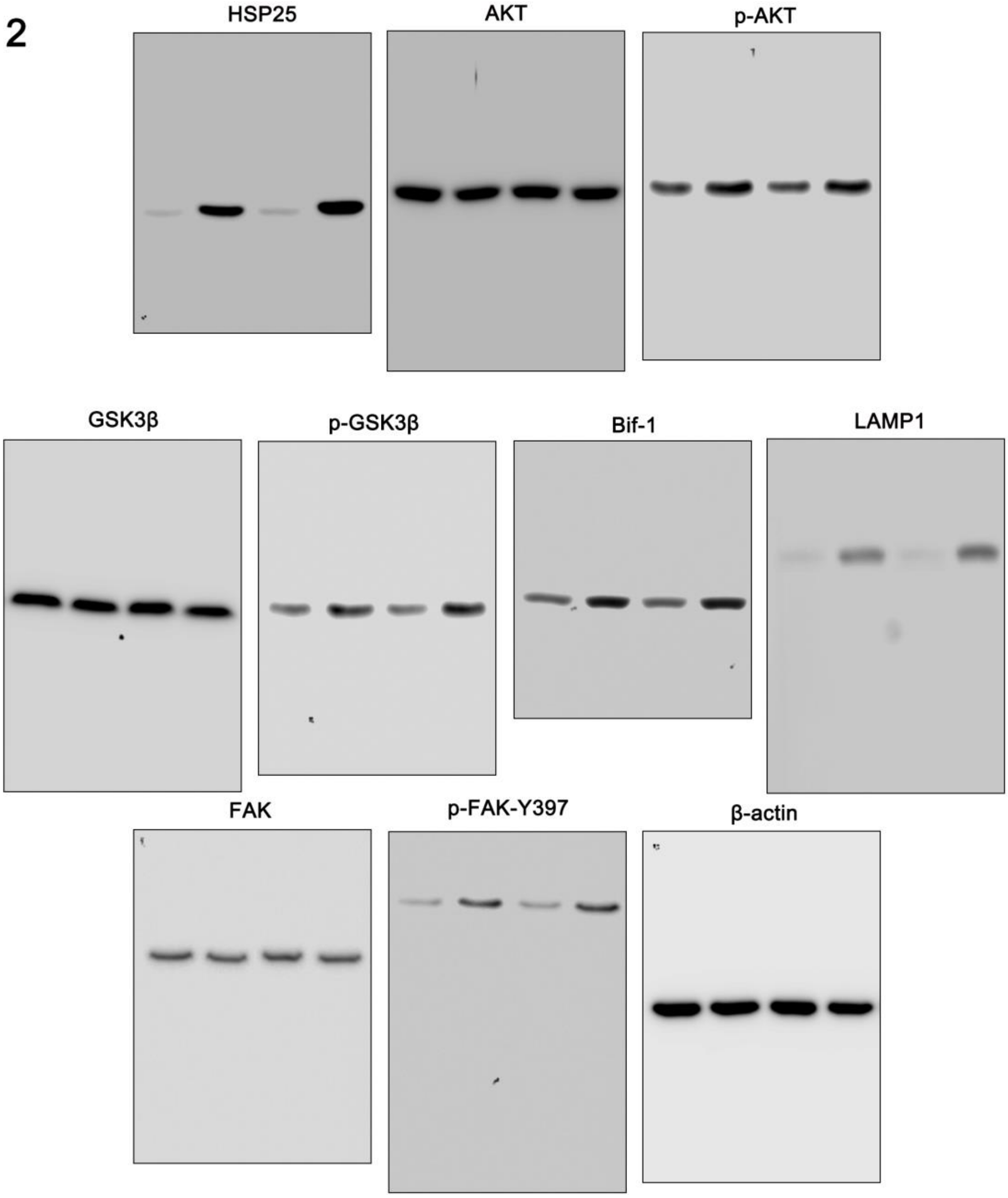

Supplementary Figure 2. Full-length gel images of Western blot data in Fig. 2.

Fig. 3

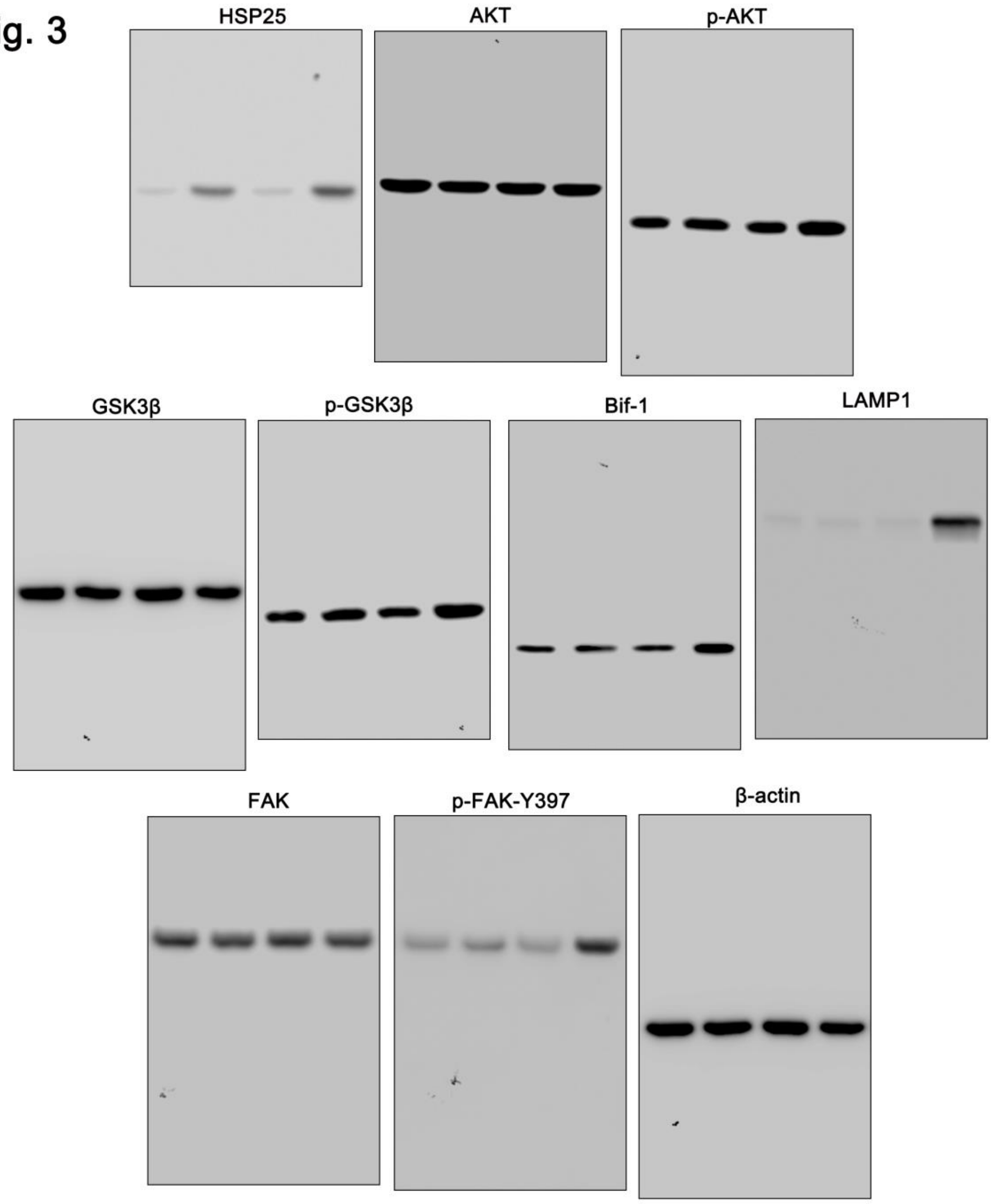

Supplementary Figure 3. Full-length gel images of Western blot data in Fig. 3.

Fig. 4

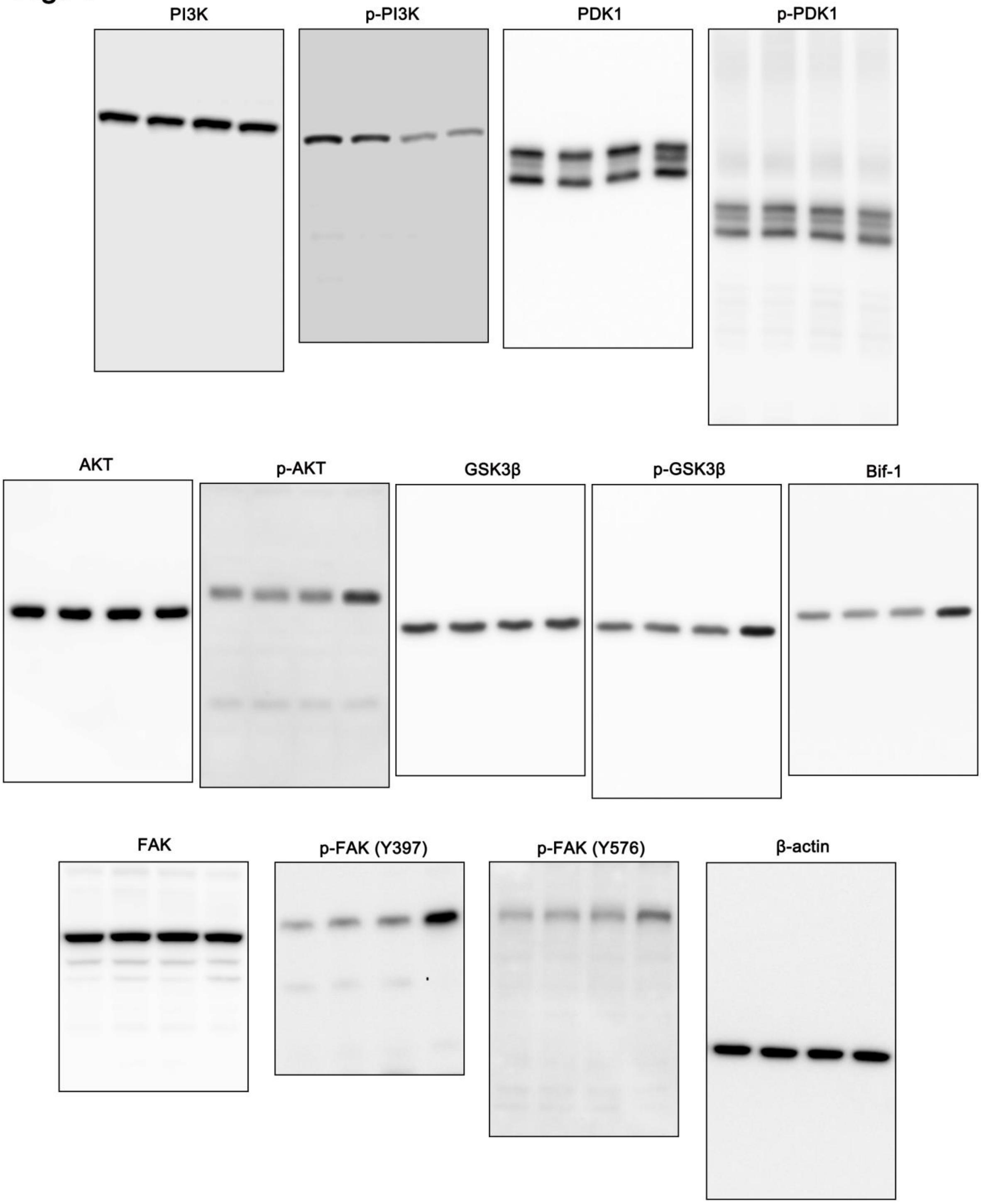

Supplementary Figure 4. Full-length gel images of Western blot data in Fig. 4.

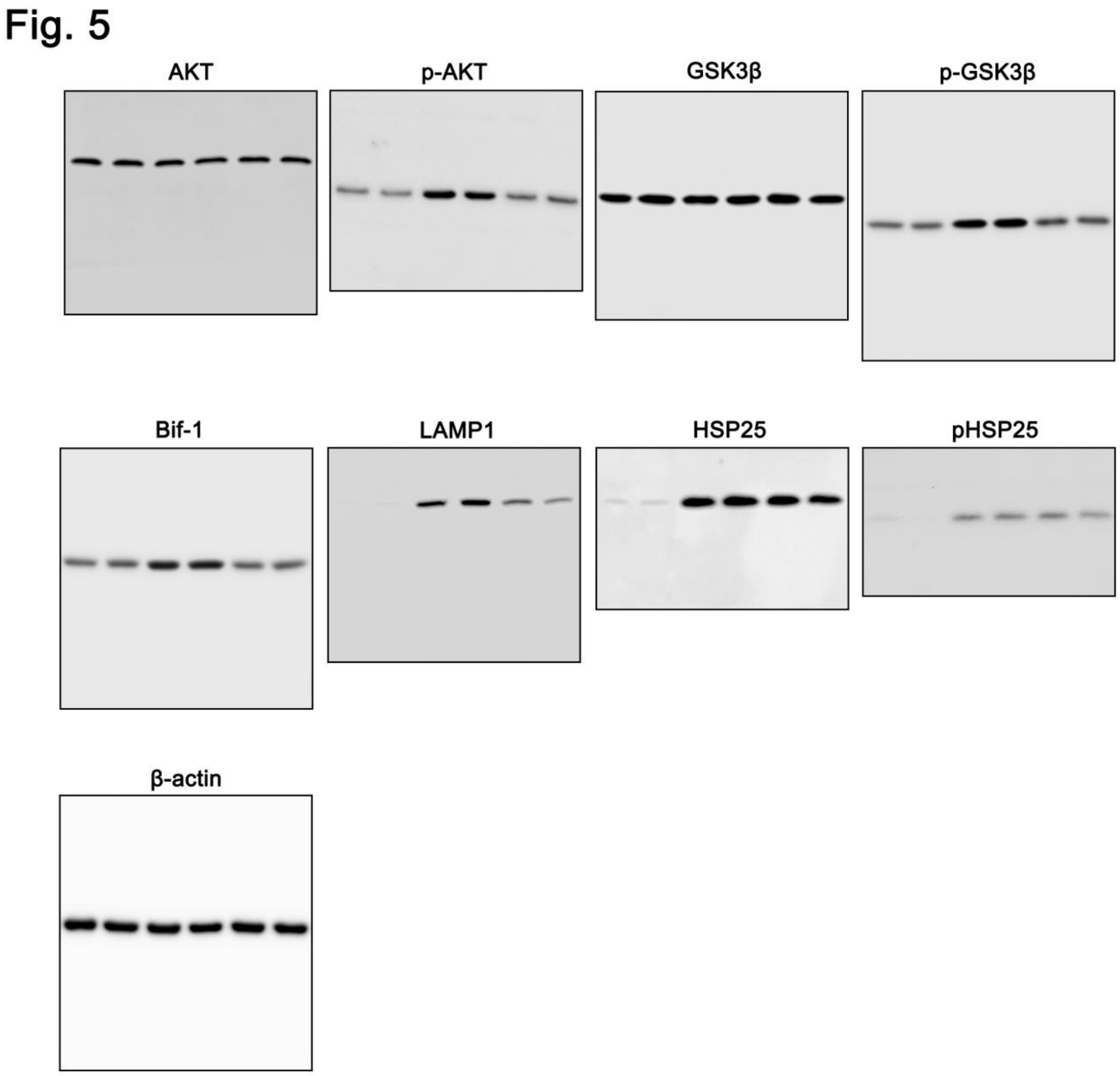

Supplementary Figure 5. Full-length gel images of Western blot data in Fig. 5.

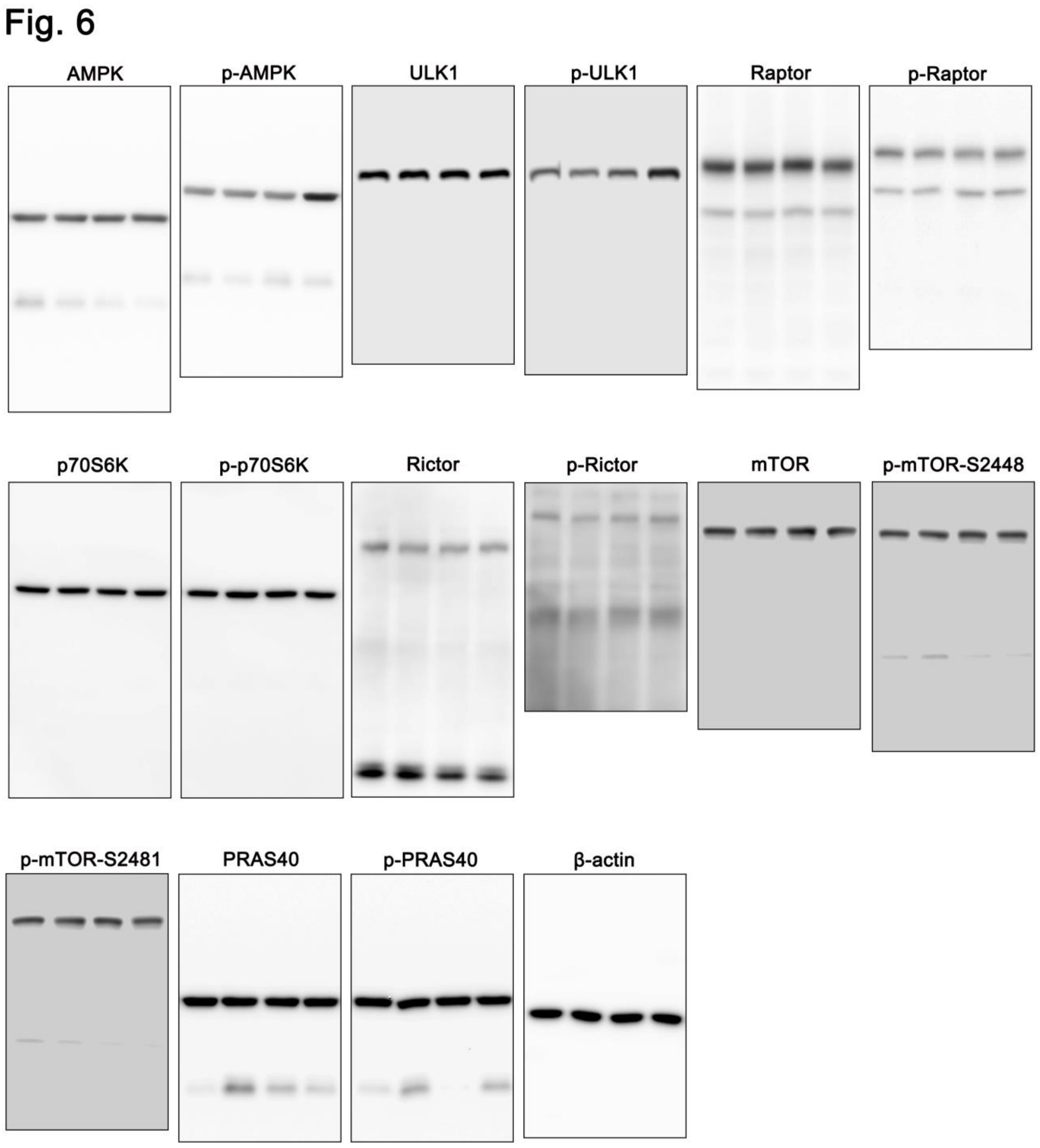

Supplementary Figure 6. Full-length gel images of Western blot data in Fig. 6.

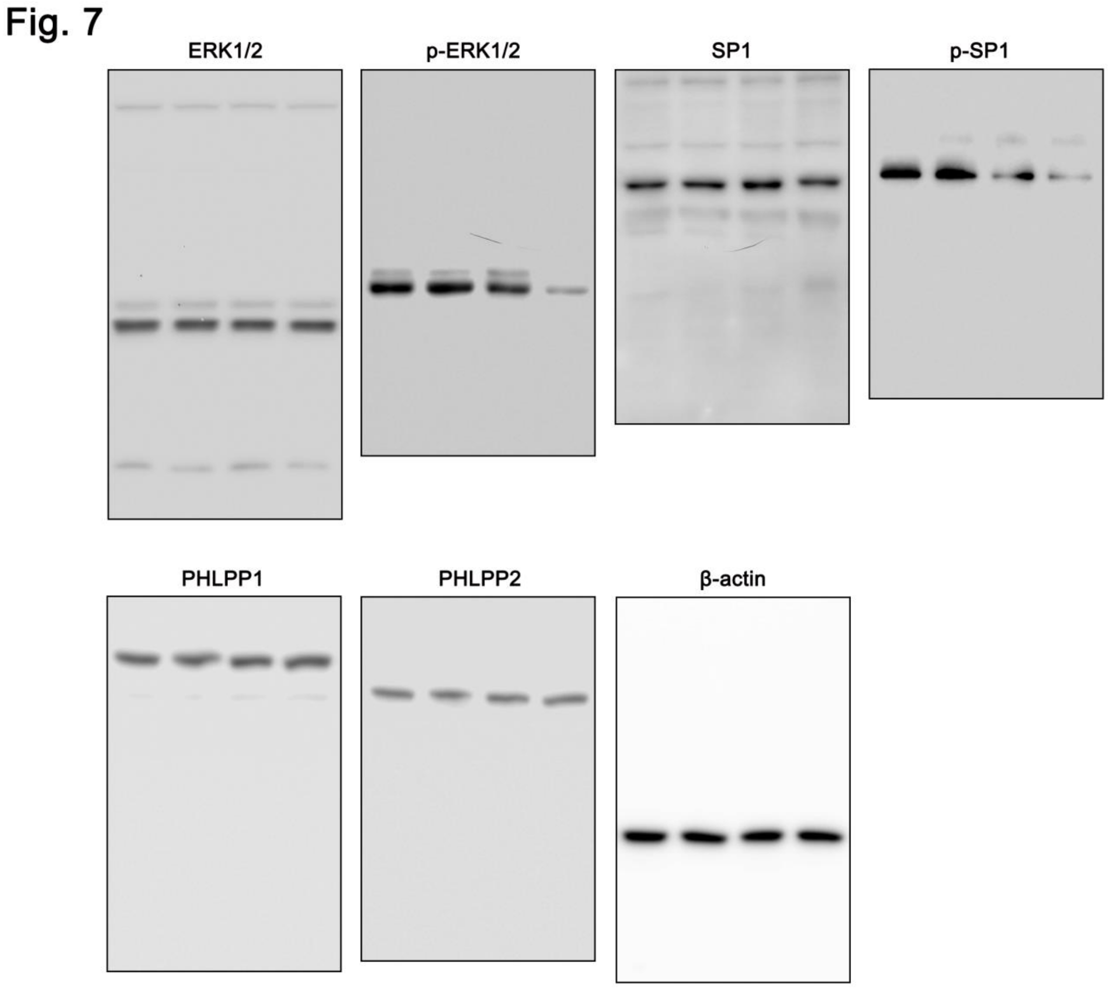

Supplementary Figure 7. Full-length gel images of Western blot data in Fig. 7.

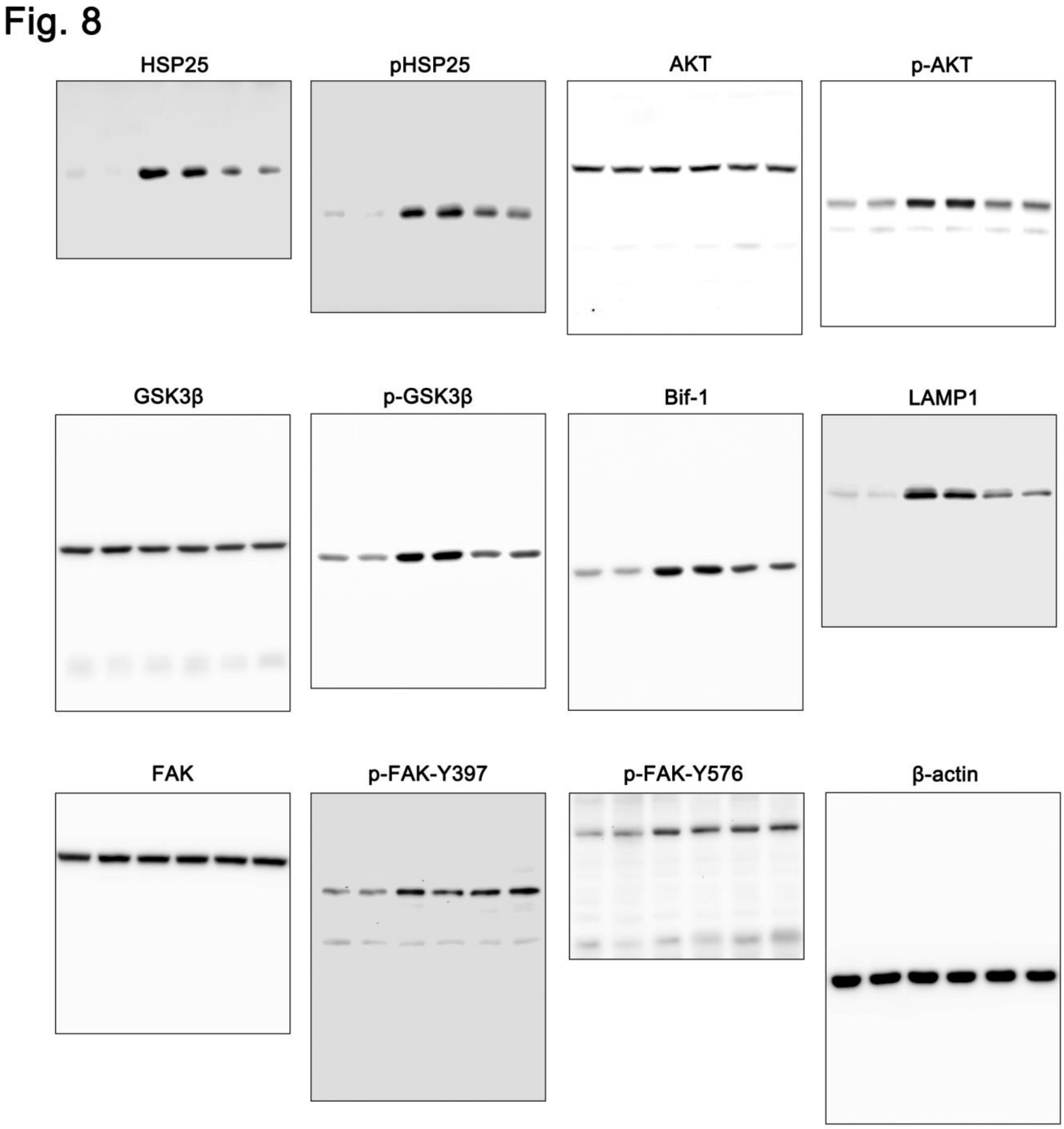

Supplementary Figure 8. Full-length gel images of Western blot data in Fig. 8.

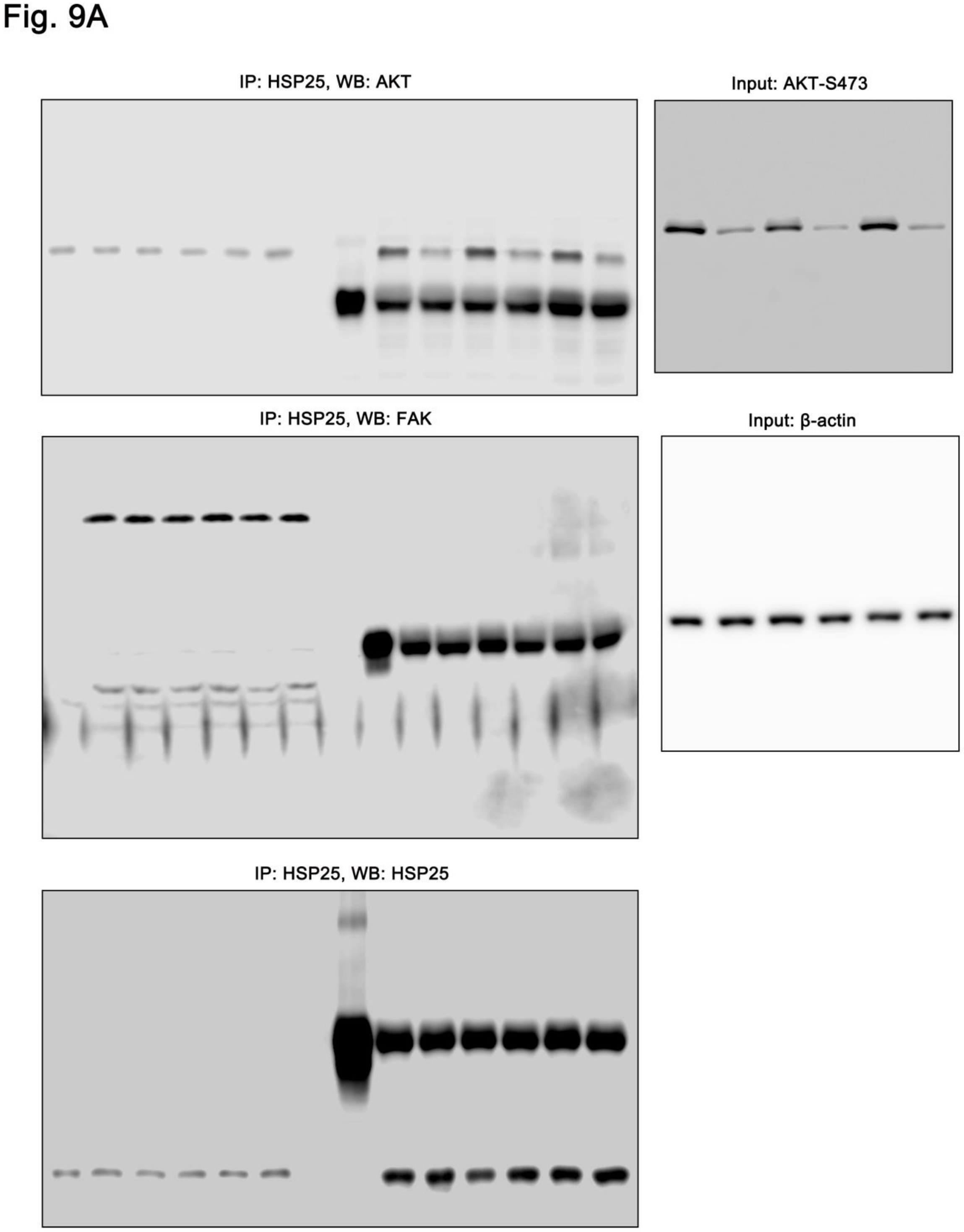

Supplementary Figure 9. Full-length gel images of Western blot data in Fig. 9A.

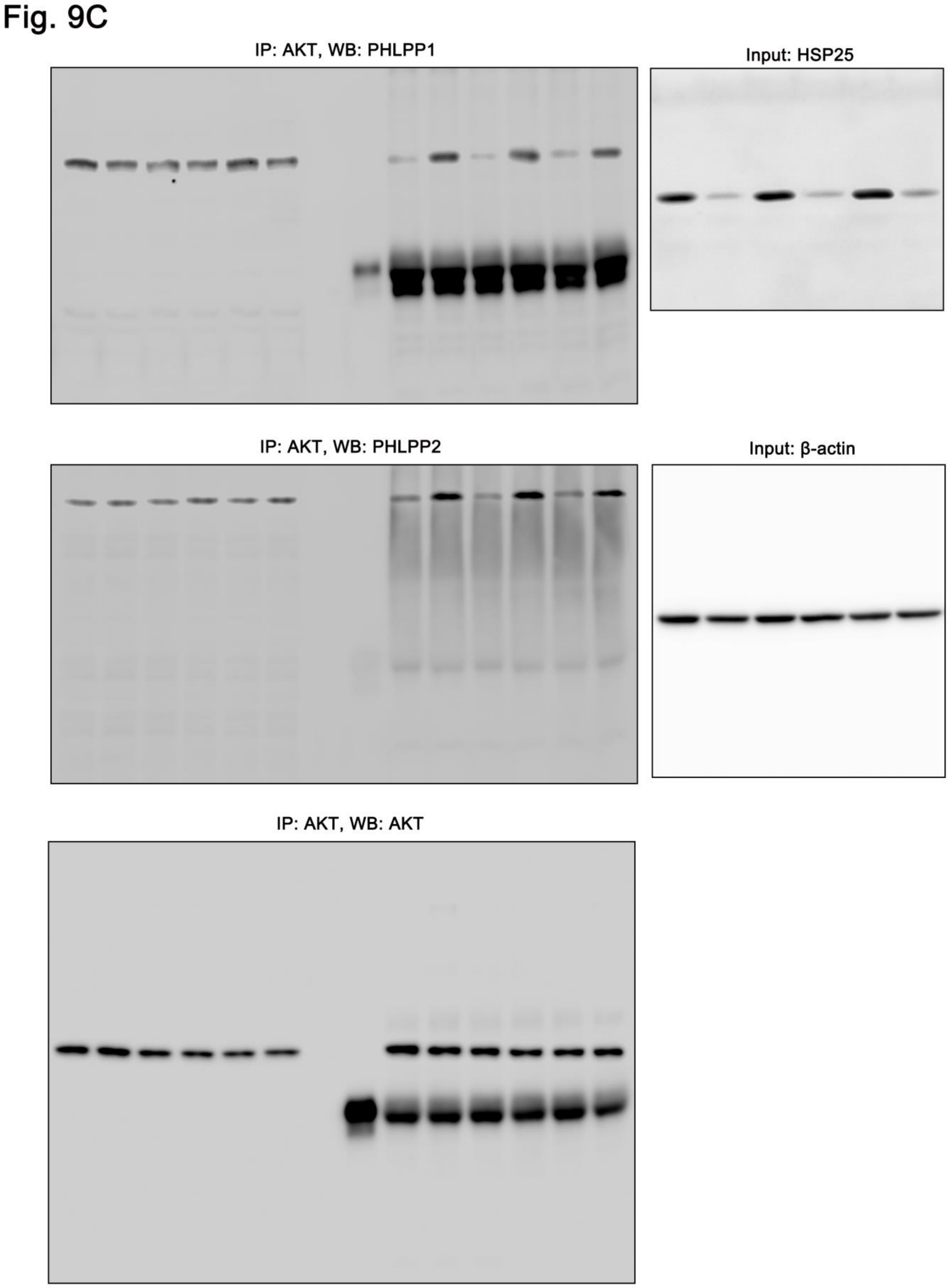

Supplementary Figure 10. Full-length gel images of Western blot data in Fig. 9C.
